# Contribution of Various Carbon Sources Toward Isoprene Biosynthesis in Poplar Leaves Mediated by Altered Atmospheric CO_2_ Concentrations

**DOI:** 10.1371/journal.pone.0032387

**Published:** 2012-02-23

**Authors:** Amy M. Trowbridge, Dolores Asensio, Allyson S. D. Eller, Danielle A. Way, Michael J. Wilkinson, Jörg-Peter Schnitzler, Robert B. Jackson, Russell K. Monson

**Affiliations:** 1 Department of Ecology and Evolutionary Biology, University of Colorado, Boulder, Colorado, United States of America; 2 Cooperative Institute for Research in Environmental Sciences, University of Colorado, Boulder, Colorado, United States of America; 3 Research Unit Environmental Simulation, Institute of Biochemical Plant Pathology, Helmholtz Zentrum München, Neuherberg, Germany; 4 Department of Biology, Nicholas School of the Environment, Duke University, Durham, North Carolina, United States of America; University of Oxford, United Kingdom

## Abstract

Biogenically released isoprene plays important roles in both tropospheric photochemistry and plant metabolism. We performed a ^13^CO_2_-labeling study using proton-transfer-reaction mass spectrometry (PTR-MS) to examine the kinetics of recently assimilated photosynthate into isoprene emitted from poplar (*Populus × canescens*) trees grown and measured at different atmospheric CO_2_ concentrations. This is the first study to explicitly consider the effects of altered atmospheric CO_2_ concentration on carbon partitioning to isoprene biosynthesis. We studied changes in the proportion of labeled carbon as a function of time in two mass fragments, M41^+^, which represents, in part, substrate derived from pyruvate, and M69^+^, which represents the whole unlabeled isoprene molecule. We observed a trend of slower ^13^C incorporation into isoprene carbon derived from pyruvate, consistent with the previously hypothesized origin of chloroplastic pyruvate from cytosolic phosph*enol*pyruvate (PEP). Trees grown under sub-ambient CO_2_ (190 ppmv) had rates of isoprene emission and rates of labeling of M41^+^ and M69^+^ that were nearly twice those observed in trees grown under elevated CO_2_ (590 ppmv). However, they also demonstrated the lowest proportion of completely labeled isoprene molecules. These results suggest that under reduced atmospheric CO_2_ availability, more carbon from stored/older carbon sources is involved in isoprene biosynthesis, and this carbon most likely enters the isoprene biosynthesis pathway through the pyruvate substrate. We offer direct evidence that extra-chloroplastic rather than chloroplastic carbon sources are mobilized to increase the availability of pyruvate required to up-regulate the isoprene biosynthesis pathway when trees are grown under sub-ambient CO_2_.

## Introduction

Isoprene (C_5_H_8_, 2-methyl-1,3-butadiene) is a principal and highly volatile biogenic hydrocarbon that is released into the atmosphere predominantly by plants [1]–[4]. Because of isoprene's reactivity with tropospheric oxidants and large global emission rate, considerable research has gone into identifying the biochemical processes that control isoprene emissions from leaves, including their sensitivities to environmental change and representation in regional and global emission models [5]–[11]. Briefly, isoprene is synthesized in leaf chloroplasts from dimethylallyl diphosphate (DMADP), a product of the deoxyxylulose-5-phosphate/2-methyl-3-buten-2-ol (DOXP/MEP) pathway, which utilizes glyceraldehyde-3-phosphate (G3P) and pyruvate (Pyr) as initial substrates [12], [13]. Both G3P and Pyr are derived from photosynthetically assimilated CO_2_, with G3P being a direct product of the reductive pentose phosphate pathway in chloroplasts. Most evidence to date indicates that pyruvate, however, is produced from photosynthate that is exported from the chloroplast, converted to phospho*enol*pyruvate (PEP) through glycolysis in the cytosol, and then imported back into the chloroplast, likely via a phopho*enol*pyruvate/phosphate translocator (PPT) [14]. While PEP transport into chloroplasts has not been directly observed, observed affinities for PEP and inorganic phosphate (Pi) of the isolated PEP-Pi Transporter (PPT) from chloroplast envelopes, and lack of a glycolytic sequence capable of converting hexose-phosphates into PEP within the chloroplast, have led to the inference that PEP is imported into C3 chloroplasts from the cytosol [15]. Once in the chloroplast, PEP is converted to Pyr by pyruvate kinase [14], [16]. Because of its direct connection to photosynthetic CO_2_ assimilation, especially through the use of G3P as a primary substrate, labeling of photosynthetic products with ^13^CO_2_ causes the ^13^C isotope to appear rapidly in emitted isoprene [17].

One mystery yet to be fully resolved, however, is why a significant fraction (∼20% on average) of emitted isoprene carbon remains unlabeled with ^13^C even after several hours of exposure to ^13^CO_2_ [18]. Using poplar leaves, Schnitzler et al. (2004) showed that alternative sources of carbon substrate exist for isoprene production in poplars, including compounds transported as carbohydrates in the xylem stream (potentially from stored carbon in roots) and starch stored in chloroplasts [19]. Also using poplar leaves, researchers have observed an increase in the fraction of unlabeled isoprene during high-temperature or severe water stress, and attributed this to greater reliance on ‘stored’ carbon, perhaps from extra-chloroplastic sources, during periods when photosynthesis rates decrease [20], [21]. Thus, there is clear evidence for the use of stored ‘older’ carbon to support isoprene biosynthesis, and there is also evidence that plants adjust their reliance on this older carbon depending on environmental conditions.

One environmental condition that has been shown to influence isoprene emission rate, especially from poplar leaves, is the atmospheric CO_2_ concentration at which the plants are grown and measured [10], [22]–[24]. Previous studies have shown a negative correlation between photosynthetic rate and isoprene emission rate when exposed to altered CO_2_ concentrations. A number of studies have demonstrated that while photosynthesis rates increase with increasing [CO_2_], isoprene emissions decrease [23], [25]. Isoprene production is rarely limited by carbon assimilated by photosynthesis; net CO_2_ assimilation fluxes on the scale of µmol m^−2^ s^−1^ are more than enough to sustain isoprene emissions typically reported on the scale of nmol m^−2^ s^−1^. This suggests that isoprene emission rates are affected via CO_2_ concentrations altering DMADP substrate availability and/or isoprene synthase activity or quantity. The biochemical mechanism responsible for this effect has not been fully resolved, but research suggests that competition between cytosolic and chloroplastic processes for available PEP substrate plays a role, with an increase in atmospheric CO_2_ concentration shifting competition in favor of cytosolic processes [24], [26], [27]. Supporting this hypothesis, Possell & Hewitt (2011) recently demonstrated an increase in PEP carboxylase activity with increasing CO_2_ concentrations and a concomitant decline in DMADP content. While the same PEP carboxylase activity levels were exhibited by trees grown under sub-ambient CO_2_ as those grown under normal, ambient CO_2_, a decreased demand for cytosolic PEP under these conditions may still result in a flux of carbon into the chloroplast [28]. Furthermore, they showed that an application of fosmidomycin (a competitive substrate inhibitor of the second enzymatic step in the MEP pathway) to plants grown under sub-ambient and elevated CO_2_ conditions resulted in isoprene emission rates that are statistically similar to those grown under ambient CO_2_ conditions. Together, these data support the hypothesis that differences in DMADP biosynthesis rate observed among CO_2_ treatments are due to changes in pyruvate and G3P availability for the MEP pathway. However, there remains much to discover about the biosynthetic kinetics and mechanisms by which G3P and Pyr are utilized for isoprene production.

One way to understand how changes in atmospheric CO_2_ affect isoprene biosynthesis is to evaluate the contribution of different carbon sources to competing metabolic processes under various CO_2_ regimes. Studies capable of resolving ^13^CO_2_ labeling kinetics, and the movement of ^13^C through precursor pools to isoprene biosynthesis, were improved considerably by the development of the proton-transfer-reaction mass spectrometer (PTR-MS). The PTR-MS approach, which allows for near-continuous measurement of compound masses in air flowing through a leaf gas-exchange chamber [18], [19], [29], was further improved by the discovery that increasing the electric field within the drift tube led to unique compound fragmentation patterns (for more detail see *Materials and methods*). These patterns increased the potential to observe changes in ^13^C labeling of a specific fragment (M41^+^) that was confirmed to be the 3-carbon methyl-vinyl fragment of the 5-carbon isoprene molecule [18]. The methyl-vinyl fragment contains two carbons contributed to isoprene biosynthesis from the Pyr substrate and one carbon from G3P. Thus, use of the PTR-MS makes it possible to track the labeling kinetics of not only the whole isoprene molecule, but also the fragment that contributes carbon from the labeled Pyr pool. We used this approach to study ^13^C labeling dynamics in the leaves of poplar trees grown and measured under different CO_2_ environments (190 ppmv, 400 ppmv, and 590 ppmv). Our goal was to: 1) resolve labeling kinetics in the whole isoprene molecule, the methyl-vinyl fragment, and, by inference, leaf pyruvate pools, to determine if different CO_2_ growth conditions influence the use of specific carbon sources for isoprene biosynthesis; and 2) to elucidate the potential pathway through which the flow of carbon from these various sources is ultimately incorporated into isoprene via pyruvate as opposed to G3P substrate.

## Materials and Methods

### Plant material and growth conditions

We grew hybrid poplar trees (*Populus × canescens*; syn. *Populus tremula* × *P. alba*) in Germany from cuttings that were initially grown in small pots with sterile sand. The misting rooms were circulated with ambient air at 24°C and maintained a photoperiod of 14 h at a PPFD of 200 µmol m^−2^ s^−1^ and 70% relative humidity (RH). Once roots formed, plants were shipped to the Duke University Phytotron, transferred to pots filled with 1∶1∶1 (v∶v∶v) sand∶perlite∶peat, and placed in one of three growth chambers (Model M-13, Environmental Growth Chambers, Chagrin Falls, OH). Plants were then grown at 27∶23°C day∶night temperatures with 16∶8 day∶night photoperiods at a PPFD of 700 µmol photons m^−2^ s^−1^ at canopy level and 50% relative humidity at peak leaf area. Plants were also grown under one of three CO_2_ concentrations, 190 ppm, 400 ppm, or 590 ppm, with the diurnal range in concentration being less than 10 ppmv. The CO_2_ concentration of chamber air was measured with an infra-red gas analyzer (LiCor 6252, Lincoln, NE) every 2–5 minutes throughout the growth period. The elevated CO_2_ environment was created by injection of pure CO_2_ into the air stream as needed, whereas low CO_2_ concentrations were maintained by scrubbing the incoming air with soda lime before injecting it with CO_2_. The trees were cut to just above soil level after growth under the CO_2_ treatments for 2 months. After growing again for two months, the trees were trimmed several nodes above the soil, and then allowed to re-grow for one month prior to making measurements. We made measurements on leaves two nodes below the second trim point, one month after trimming. These leaves were estimated to be three months old and were fully expanded. The isoprene emission rates for these leaves generally ranged from 4.5–5.5 nmol m^−2^ s^−1^ when measured under ambient CO_2_ at 30°C. These rates are within the same range of isoprene emission rates observed for leaf Node 9 in the studies of Behnke et al. (2007) on the same wild-type poplar lines used in this study, and represent leaves with fully-matured isoprene emission capacity [30]. The CO_2_ treatments were rotated among the three chambers every three weeks to minimize chamber effects, and plants were moved from spot-to-spot within each chamber on a weekly basis to minimize spatial biases on growth. For this study, we used seven trees from each growth chamber, 21 plants in total.

### Leaf gas-exchange measurements

Point measurements of baseline gas exchange were made throughout the experiment using a portable photosynthesis system and a standard 6 cm^2^ leaf chamber equipped with a programmable LED light source (LI-6400, LiCor Inc., Lincoln, NE, USA). Because the Licor instrument is differentially sensitive to ^13^CO_2_ and ^12^CO_2_, it was not possible to simultaneously measure physiological responses and isoprene emission rates during labeling. Therefore, one leaf two nodes below the second trim point (one month after trimming) was measured from trees grown under elevated (590 ppm) and sub-ambient CO_2_ (190 ppm) either prior to or after the labeling experiment. These measurements were made under light-saturating conditions (1,000 µmol photons m^−2^ s^−1^) at 30°C under the growth CO_2_ conditions of the plant. Sampling occurred at three points during the day (early morning, midday, and late afternoon) to obtain a daily mean for net CO_2_ assimilation rate (A), stomatal conductance rate (g_s_), and intercellular CO_2_ concentration (C_i_). These data offer an appropriate framework in which to understand how altered CO_2_ growth conditions affect the average carbon metabolism of poplars and, subsequently, their average isoprene biosynthesis kinetics and emission rates. Separate from the PTR-MS isoprene measurements (described below), simultaneous isoprene emission rates were measured relative to the gas exchange measurements by diverting a fraction of the outgoing air from the leaf cuvette to a chemi-luminescence based fast isoprene sensor (FIS) (Hills Scientific, Boulder, CO, USA).

### Proton Transfer Reaction-Mass Spectrometry

To determine the kinetic dynamics at which ^13^C progressively replaced ^12^C in the isoprene molecule and its fragment, we combined a LiCor 6400 cuvette system (LiCor Inc., Lincoln, NE, USA) with a PTR-MS (Ionicon GmbH, Innsbruck, Austria). This unique system was used to determine the isoprene concentration in the outgoing cuvette air and the mass variants (isotopomers) of isoprene and associated fragments, which reflected the time-dependent turnover of ^12^C after labeling the air with ^13^CO_2_.

One leaf (2 nodes below the trim point) per individual was placed in a LI-6400 portable photosynthesis system leaf chamber as described above (LiCor, Inc., Lincoln, Nebraska). The flow of air through the cuvette was 350 ml min^−1^, which was sufficient to allow for a reasonable ‘wash-in’ and ‘wash-out’ time during the labeling experiment. Approximately 50 ml min^−1^ of cuvette air was diverted to the PTR-MS using Teflon tubing. Inlet air to the cuvette was obtained from an air source that was mixed each day using a clean air generator (model 737, Aadco Inc., Cleves, OH, USA) with an activated charcoal scrubber on the outlet to ensure air purity, and the addition of either ^12^CO_2_ (Airgas, Inc., Durham, NC) or ^13^CO_2_ (Cambridge Isotope Laboratories, Inc., Andover, MA) through manual injection. The volume of each unlabeled or labeled CO_2_ source gas required to create the appropriate CO_2_ concentration (v/v) for each treatment was calculated for 190 ppm, 400 ppm, and 590 ppm assuming 90 L of VOC (volatile organic compound)-free air. Depending on the treatment, either 17.1 mL, 36 mL, or 53.1 mL, respectively, of source ^12^CO_2_ or ^13^CO_2_ were injected into an empty 100 L Tedlar bag (CEL Scientific Corp, Santa Fe Springs, CA), and VOC-free air was immediately pumped in at 4.5 L/min for 20 minutes. This mixed air source was then stored in the large Tedlar bag, and slowly evacuated as needed for the experiment. Positive pressure was maintained in the cuvette to prevent the ingress of contaminants. During the measurement period, leaves in the cuvette were maintained at 30°C leaf temperature, 1000 µmol m^−2^ s^−1^ photosynthetically active radiation (PAR) and 50–70% relative humidity (RH). In each experiment, leaves in the cuvette were fed air with ^12^CO_2_ at the same concentration as their growth environment until a stable isoprene emission rate was observed. Once a stable point was reached, the air source was switched to ^13^CO_2_ at the same concentration, and time-dependent changes in isoprene mass variants were monitored. Once all labeled isoprene mass variants/signals were stable, the ^13^CO_2_ supply was switched back to ^12^CO_2_. Due to complications with differential sensitivity of the LiCor CO_2_ sensor to ^13^CO_2_ and ^12^CO_2_, we were not able to measure the ^13^CO_2_ concentration of the air in the Tedlar bag, which was used as the labeling source. However, it was mixed to specifications to provide 190 ppm, 400 ppm, and 590 ppm CO_2_, respectively, and we did check the ^12^CO_2_ concentration in the chamber air before and after the labeling to confirm that it remained within 20 ppmv of the target values.

The PTR-MS instrument design and underlying principles of operation have been described previously in detail [31]. For this study, the instrument was operated at an E/N of 140 Td to induce a high degree of fragmentation. Operating the instrument under these conditions changed the mass spectrum to favor higher production frequencies of the 3-C fragment from isoprene measured at M41^+^ (32.6%) and M42^+^ (1.1%), as opposed to lower frequencies observed under normal operating conditions that produce 7.1% and 0.2% M41^+^ and M42^+^, respectively [18]. The drift tube pressure, temperature, and voltage were 1.96 hPa, 60°C, and 550 V, respectively. The count rate of H_3_O^+^H_2_O ions measured before labeling was less than 1% of the count rate of H_3_O^+^ ions, which was 9.2–10.9×10^6^ counts s^−1^. The PTR-MS was calibrated by generating a standard curve for both M41^+^ and M69^+^ after measuring the counts per second (cps) of these masses with different known isoprene concentrations, which were created by dilution of a 1.5 ppmv isoprene standard (Scott-Marrin, Inc., Riverside, CA) with humidified VOC-free air at the beginning of each day's experiment. The detection limit of the PTR-MS based on the calibration of m/z 69^+^ was 55 ppt/normalized count per second. Leaf isoprene fluxes were calculated as:
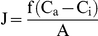
Where f is the flow rate through the cuvette (mL min^−1^) and A is the leaf area enclosed within the cuvette (cm^2^). C_a_−C_i_ is the difference in gas partial pressure between the empty and leaf-filled cuvette, expressed in nmol mol^−1^.

### Calculations and Statistical Analysis

To compare the labeling dynamics of both the 3-carbon fragment, M41^+^, and the 5-carbon parent molecule, M69^+^, we measured the time dependent change in the proportion of ^13^C simultaneously incorporated into each. To do this we first calculated the total labeled carbon atoms (^13^C) (on a molar basis) in both M41^+^ and M69^+^ at each point in time, and then determined the total isoprene emission rate for each point in time. Total labeled carbons were determined for the methyl-vinyl fragment (M41^+^) by summing the products of the mass variants' emission rates (M42^+^, M43^+^, and M44^+^) and the number of labeled carbons represented by their detection (1, 2, and 3, respectively). For example, at any point in time the number of labeled carbons in the M41^+^ 3-C methyl-vinyl fragment was equal to {(M42^+^)+(M43^+^ 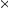
2)+(M44^+^ 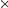
3)}, whereas the number of labeled carbons in the 5-C parent molecule was equal to {(M70^+^)+(M71^+^ 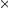
2)+(M72^+^ 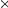
3)+(M73^+^ 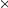
4)+(74^+^ 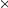
5)}. The total labeled carbon was then divided by the total isoprene emission rate at each point during the experiment, both before and after labeling, to obtain and compare the number of labeled carbons in both the fragment and parent molecule simultaneously through time. The number of labeled carbons in both the parent molecule and the fragment were graphed together over time, again both before and after labeling, but separately for each treatment. Results were based on a qualitative assessment of how the lines diverged in time relative to the number of labeled carbons plotted on the y-axis. If labeled carbon (^13^C) was added to the parent molecule (M69^+^) through the methyl-vinyl fragment (M41^+^), then both lines would increase simultaneously. If labeled carbon was being incorporated into the parent molecule faster than it is appearing in the fragment, a divergence of the M41^+^ line from the M69^+^ would result.

To evaluate the effects of different [CO_2_] on the labeling rate of whole-isoprene and the M41^+^ fragment (representing the substrate originating from pyruvate), we had to account for the simultaneous gain and loss of labeled carbons (^13^C) as they successively moved through the observed mass fractions. Conceptually, this can be accomplished by considering the rates of change of each isotopomer in terms of the “loss” of carbon from the mass preceding it. For example, as M72^+^ gained a labeled carbon and became M73^+^, this subsequently caused an equal “loss” of M72^+^. Observing the labeling of M72^+^ graphically over time then shows a positive slope (representing the “gain” of ^13^C into M71^+^, thus producing M72^+^) followed by a peak and a subsequent shorter negative slope (denoting the “loss” or movement of ^13^C into the higher mass, in this case M73^+^). With the data expressed in its raw form exhibiting two slopes as described, it would be extremely difficult to obtain an accurate rate of labeling for this mass variant. Therefore, to accurately account for movement of ^13^C between masses, the slopes, or rates of ^13^C gain and loss for each mass from the start of labeling until the concentration of all masses reached steady state in the air leaving the cuvette, were summed (denoted by an “s” to distinguish from individual masses) according to: sM70^+^ = (M70^+^+M71^+^+M72^+^+M73^+^+M74^+^), which represents the rate of labeling of isoprene where isoprene has at least 1 carbon labeled; sM71^+^ = (M71^+^+M72^+^+M73^+^+M74^+^), representing the rate of labeling of isoprene molecules that have at least 2 carbons labeled; and sM72^+^ = (M72^+^+M73^+^+M74^+^), representing the rate of labeling of isoprene molecules that have at least 3 carbons labeled, and so on. The same calculations were applied to the fragment M41^+^ where sM42^+^ = (M42^+^+M43^+^+M44^+^), representing the rate of labeling of isoprene's fragment where at least one carbon on this 3-C subunit is labeled; and sM43^+^ = (M43^+^+M44^+^), representing the rate of labeling of the 3-C subunit that have at least 2 carbons labeled, and so on. The slopes of each individual signal (expressed as molecules/cycle, where each cycle represents every 30 seconds and a PTR-MS dwell time of 2 seconds) were calculated using a generalized linear model with an identity link function that provides the relationship between the linear predictor and the mean of the distribution function, which in this case is normal. The effect of the three different growth and measurement CO_2_ concentrations on the rate of labeling of each analog was then analyzed using a one-way analysis of variance (ANOVA).

To determine the proportion of each analog of both the parent isoprene molecule and the methyl-vinyl fragment that was labeled at the end of the experiment, the emissions for each analog at each point during the last 30 minutes of the total 1.5–2 hour labeling time (once the mass had become stable and reached its maximum labeling) were divided by the total emission rate at that same point in time (sum (M69^+^ through M74^+^)). A one-way ANOVA was performed to determine if proportions of labeling (in both the parent and fragment molecules) differed for each analog between treatments. Isoprene emission rates for individuals were calculated by averaging the steady-state emission of M69^+^ during exposure of the leaf to ^12^CO_2_ prior to the ^13^CO_2_ labeling. A one-way ANOVA was used to evaluate differences in isoprene emissions between treatments. A Student's *t* test was used to determine differences in net CO_2_ assimilation rates (A), stomatal conductance rates (g_s_), and intercellular CO_2_ concentrations (C_i_) between trees grown under sub-ambient and elevated CO_2_ conditions. Finally, linear regression models were used to evaluate the relationship between isoprene emission rate and each of the physiological parameters described above. All statistical analyses were performed with R (2.10.1, Vienna, Austria).

## Results

### Physiological data and total isoprene emission rates

**Figure 1:**
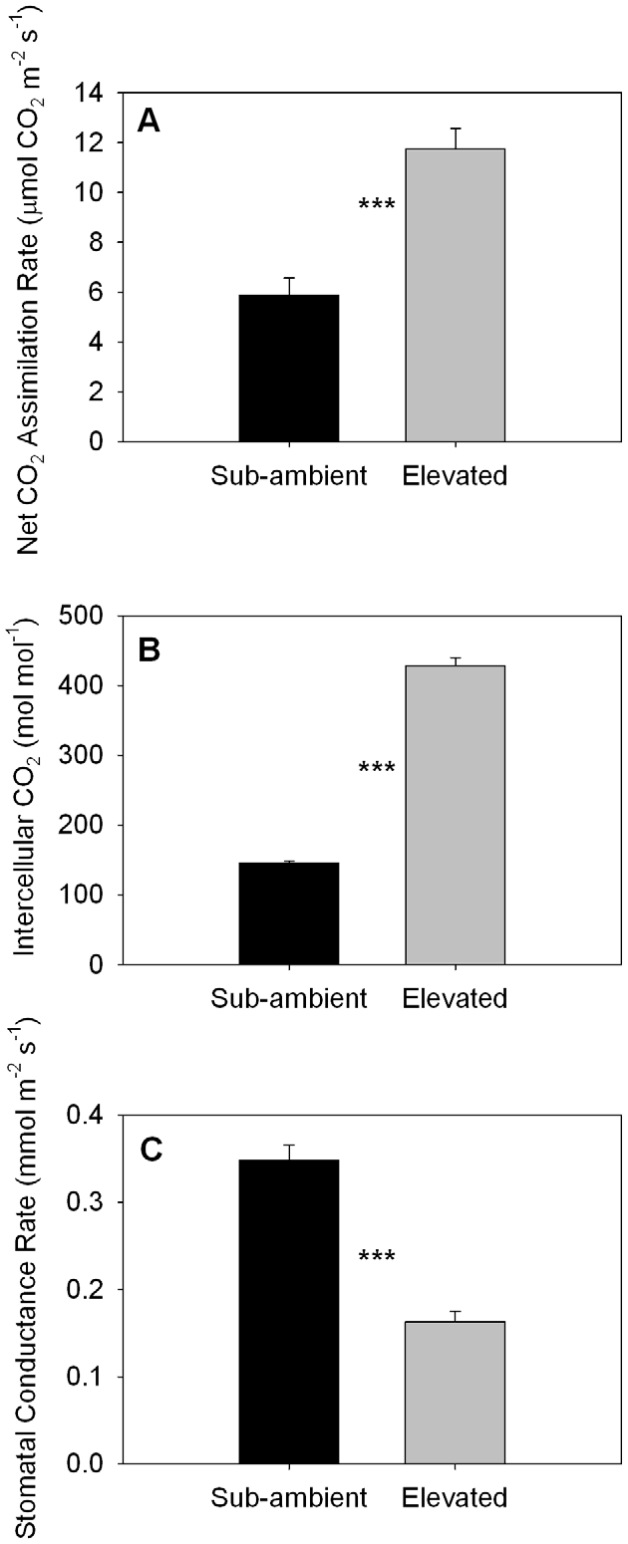
Photosynthesis, intercellular CO_2_, and stomatal conductance for poplars grown under sub-ambient and elevated CO_2_ regimes. Net CO_2_ assimilation rate (A), intercellular CO_2_ concentration (B), and stomatal conductance rate (C) for trees grown under sub-ambient (190 ppm) and elevated (590 ppm) CO_2_ conditions prior to ^13^CO_2_ labeling experiment. Error bars represent the standard errors of the mean (SEM) and means with (***) are significantly different (*P*≤0.0001). Trees grown under elevated CO_2_ exhibited significantly higher intercellular CO_2_ concentrations and net CO_2_ assimilation rates relative to poplars grown under sub-ambient CO_2_ conditions. Conversely, trees exposed to elevated CO_2_ growth conditions demonstrated significantly lower stomatal conductance rates compared to those grown under sub-ambient CO_2_.

Leaf gas exchange measurements were made concurrently with isoprene emission rates from trees grown under sub-ambient and elevated CO_2_ conditions. Trees grown under elevated CO_2_ had significantly higher net CO_2_ assimilation rates (11.74±0.88µmol m^−2^ s^−1^) and intercellular CO_2_ concentrations (428.11±11.52 µmol mol^−1^) than trees grown under sub-ambient CO_2_ conditions (5.88±0.66 µmol m^−2^ s^−1^ and 145.5±3.12µmol mol^−1^, respectively; n = 6 and *P*<0.0001) (Figure 1A and B). It should be noted that all values, unless otherwise specified, are reported as the mean ± standard error of the mean, sample size, and probability of Type I error, respectively. Furthermore, trees grown under elevated CO_2_ exhibited significantly lower stomatal conductance rates (0.162±0.019 mol m^−2^ s^−1^) compared to those grown under sub-ambient CO_2_ (0.348±0.162 mol m^−2^ s^−1^; n = 6, *P*<0.0001) (Figure 1C). We used linear regression models to evaluate whether significant relationships exist between each of the three physiological parameters described above and total isoprene emission rates, which were measured simultaneously. Results indicated a significant *positive* linear relationship between stomatal conductance and isoprene emission rate (R^2^ = 0.58; *P*<0.0001) and a significant *negative* linear relationship between intercellular CO_2_ concentration and isoprene emission rate (R^2^ = 0.64; *P*<0.0001) as well as between net CO_2_ assimilation rate and isoprene emission rate (R^2^ = 0.18; *P* = 0.006). Individual regressions were performed on all data for each continuous variable (i.e. after combining values from trees grown under both sub-ambient and elevated CO_2_ conditions).

### Qualitative analysis of recently assimilated carbon incorporation into isoprene

**Figure 2:**
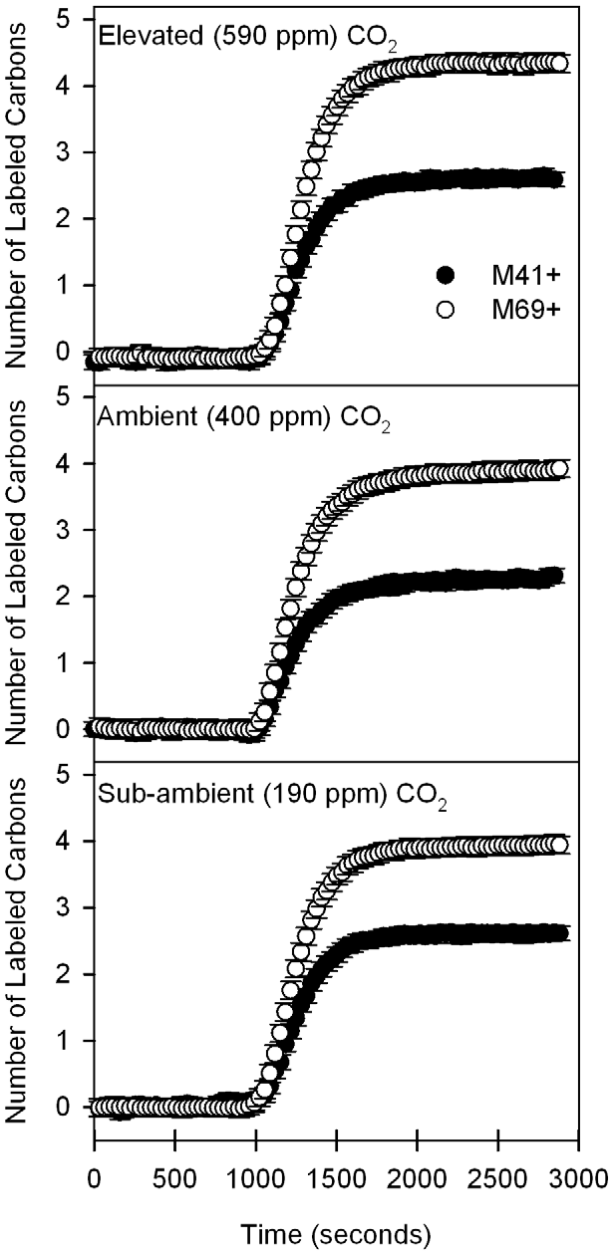
^13^CO_2_ labeling of M41^+^ and M69^+^ as a function of CO_2_ concentration and time. The number of labeled carbons present in both the M41^+^ fragment and M69^+^ parent isoprene molecule prior to and after ^13^C labeling with error bars representing the standard error of the mean (SEM). Change in the lines are evaluated in reference to the number of labeled carbons in both the fragment and parent molecule over time, with lines falling on one another representing labeling occurring simultaneously in both molecules and a divergence representing a faster label incorporated into M69^+^ that is not derived from M41^+^. Before leaves were exposed to ^13^CO_2_ labeling at 1000 seconds, plants were exposed to the same ^12^CO_2_ concentrations at which they were grown. As expected, no labeling occurred for either M41^+^ (closed circles) nor M69^+^ (open circles) before labeling. Immediately after labeling, one carbon was labeled in both the parent molecule and the fragment (demonstrated by the simultaneous increase in both lines), suggesting that the first carbon used to synthesize isoprene is contributed from the M41^+^ fragment. However, as time progressed and a second carbon becomes labeled on the parent molecule, the M41^+^ lines diverged for leaves grown in all three treatments, suggesting that the second labeled carbon on the isoprene molecule is not coming from the M41^+^ subunit.

When comparing the simultaneous change in the number of labeled carbons in both the fragment (M41^+^) and parent molecule (M69^+^) among the three CO_2_ regimes, both lines fall directly on one another, which indicated an immediate labeling of the first carbon (Fig. 2). This result was consistent with the hypothesis that the ^13^C that initially enters the isoprene pool is recovered in the 3-C methyl-vinyl fragment. Keep in mind that M41^+^ and M69^+^ were initially unlabeled compounds with respect to ^13^C. As time in the presence of ^13^CO_2_ progressed, if ^13^C was added to M69^+^ through the methyl-vinyl fragment, then both M41^+^ and M69^+^ should have increased simultaneously. While Figure 2 shows this to be the case for the labeling of the first carbon, as time progressed and a second carbon became labeled on the parent molecule, the M41^+^ lines diverged for leaves grown under all three treatments. Because the labeling happened faster for the second carbon in the isoprene molecule than for the second carbon in the methyl-vinyl fragment, it appears that second labeled carbon on the isoprene molecule was not coming from the M41^+^ subunit. This suggests relatively fast incorporation of MEP substrate derived directly from G3P rather than from pyruvate. Furthermore, the lines first *begin* to diverge most quickly for leaves from the low and ambient CO_2_ treatments (after ∼1 carbon was labeled), while the line of the high CO_2_ treatment leaves first *begin* to diverge later (after ∼1.5 carbons were labeled) (Fig. 2). This result was consistent with a faster incorporation of ^13^C into pyruvate in the leaves of plants grown under high CO_2_.

### Exposure of poplar leaves to ^13^CO_2_ elucidates the contribution of pyruvate-derived versus different carbon sources to isoprene synthesis over time

**Figure 3:**
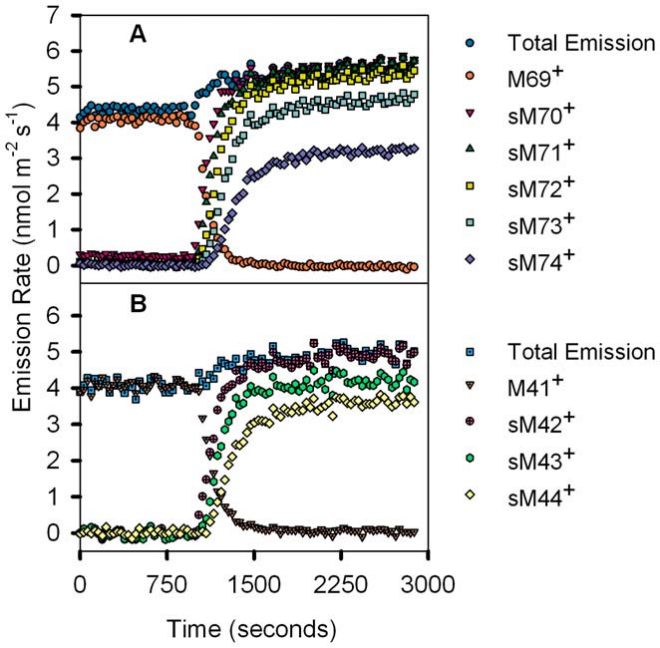
^13^CO_2_ labeling of carbon atoms in M69^+^ and M41^+^ and their isotopomers through time. (A) ^13^CO_2_ labeling of carbon atoms in trees grown and measured in ambient CO_2_ conditions (400 ppm CO_2_) in the parent isoprene molecule, as characterized by a decrease in the M69^+^ signal (orange circles) and simultaneous increase in its isotopomers (denoted as sums) as labeled carbons were successively incorporated through time. Total emission (blue circles), sM70^+^ (red downward triangles), sM71^+^ (green triangles), sM72^+^ (yellow squares), sM73^+^ (sea green squares), sM74^+^ (purple diamonds) are represented. (B) ^13^CO_2_ labeling of carbon atoms in trees grown and measured at 30°C in ambient CO_2_ conditions (400 ppm CO_2_) in the 3-C methyl-vinyl isoprene fragment, characterized by a decrease in the M41^+^ signal (light orange dotted downward triangles) with a simultaneous increase in its labeled isotopomers (denoted as sums). Total emission (blue dotted squares), sM42^+^ (pink crossed circles), sM43^+^ (green hexagons), sM44^+^ (yellow diamonds) are represented. Before leaves were exposed to ^13^CO_2_ labeling at 1000 seconds, plants were exposed to the same ^12^CO_2_ concentrations at which they were grown. The simultaneous labeling of the first carbon in the parent molecule (sM70^+^) and the fragment (sM42^+^) suggest that the first carbon contributing to the synthesis of isoprene comes from the M41^+^ fragment. However, while all of the isoprene molecules show the next two carbons labeled shortly after (sM71^+^ and sM72^+^), the next two carbons on the M41^+^ fragment (sM43^+^ and sM44^+^) are never fully labeled and may result from the incomplete labeling of pyruvate.

To examine the relative contribution of pyruvate-derived carbons for isoprene synthesis, we analyzed real-time ^13^CO_2_ labeling kinetics for both the parent isoprene molecule (M69^+^) and its 3-C fragment (M41^+^) for a representative tree grown under ambient CO_2_ of 400 ppm (Figure 3). By examining simultaneous changes between labeling in the parent molecule (Figure 3A) with changes in the 3-C fragment (Figure 3B), one could obtain a detailed account of the sequence with which labeled carbons were contributed to isoprene synthesis via the 3-C (methyl-vinyl) or 2-C fragments of the fractured isoprene molecule. From Figure 3, there was an immediate and extremely fast increase in the sM42^+^ and sM70^+^ signals following ^13^CO_2_ labeling. This result confirmed that isoprene emission rate was closely coupled to carbon assimilation and suggested that the addition of labeled carbon to the 3-C fragment had the same consequence for the signal of the parent molecule. This result also supported the conclusions from the labeled carbon data for both M41^+^ and M69^+^, which showed that the first labeled carbon transferred into the isoprene pool was recovered as part of the methyl-vinyl subunit. We note that there was a slight increase in the total isoprene emission rate and total amount of 3-C methyl-vinyl isoprene fragment detected when we switched from the ^12^CO_2_ source to the ^13^CO_2_ source. This effect was detected in all treatments, and was small in magnitude compared to the differences in total emission rates. It is possible that the CO_2_ concentration was slightly lower than desired in the ^13^CO_2_ source, but we were careful to prepare this source according to precise calculations. It is also possible that there was a small decrease in flow rate through the chamber when the sources were changed, and that the flow controller we used was differentially biased toward the presence of ^13^CO_2_.

The fast incorporation of recently assimilated carbon into isoprene via the 3-C methyl-vinyl fragment was then followed by an equally fast incorporation of a second labeled carbon, as characterized by the slope exhibited in sM71^+^ (Fig. 3A). Eventually, at least 2 labeled carbons occurred in all isoprene molecules emitted, regardless of CO_2_ growth conditions (data not shown), as also demonstrated because the sM71^+^ signal plateaus at the maximum total emission value. However, the labeling data for the sM43^+^ fragment (Fig. 3B) revealed that, at maximum labeling, not all of the molecules in the 3-C methyl-vinyl fragment had at least two ^13^C, even though there were at least 2 carbons labeled in all the parent molecules. These observations supported the conclusion that the second isoprene carbon to be labeled came from a source that was not part of the methyl-vinyl fragment containing carbon from pyruvate and presumably originated from the movement of labeled carbon into the MEP pathway through the direct incorporation of G3P substrate. Likewise, the same argument could be used to demonstrate that the third carbon contributed to isoprene biosynthesis did not originate from the methyl-vinyl fragment, indicative of pyruvate. Again, it was evident that all of the emitted isoprene eventually contained at least 3 labeled carbons, as illustrated by the sM72^+^ signal reaching the total emission plateau (Fig. 3A). Yet, not all of the 3-C methyl-vinyl fragments obtained from isoprene were completely labeled, as illustrated in the sM44^+^ signal not reaching the maximum total value (Fig. 3B).

Because we have not accounted for the 2 remaining carbons on the 3-C methyl-vinyl fragment, several lines of reasoning led us to deduce that these carbons were labeled last in the sequence of ^13^C transfers into the isoprene pool, with this ‘slow’ transfer having occurred through the pyruvate substrate pool. First, we know that two of the carbons on the 3-carbon fragment came from pyruvate. Additionally, as discussed above, the one carbon atom in this fragment that was derived from G3P was also, based on knowledge of photosynthetic metabolism, the first carbon within the G3P pool to have been labeled after the assimilation of ^13^CO_2_. Second, at the end of the experiment (after ∼2 hours), the labeling data showed that the sM43^+^ and sM44^+^ fragments never reached the pre-labeling maximum exhibited by M41^+^, despite the fact that the abundance of M41^+^ eventually went to zero. This meant that some of the 3-C methyl-vinyl fragments only had one or two ^13^C-labeled carbons, not three (Fig. 3B). On the basis of this evidence, we concluded that C-1 of the G3P molecule carried the ^13^C label through the MEP pathway and into isoprene first, followed by incorporation of ^13^C through the C-2 and C-3 carbons of G3P substrate and that any ^13^C that entered through pyruvate came later. Furthermore, it appeared that the fraction of emitted isoprene carbon that remained unlabeled, even after several hours in the presence of ^13^CO_2_, most likely originated from pyruvate carbon.

### Rates of ^13^CO_2_ labeling and the proportion of labeling at steady state between CO_2_ treatments

**Figure 4:**
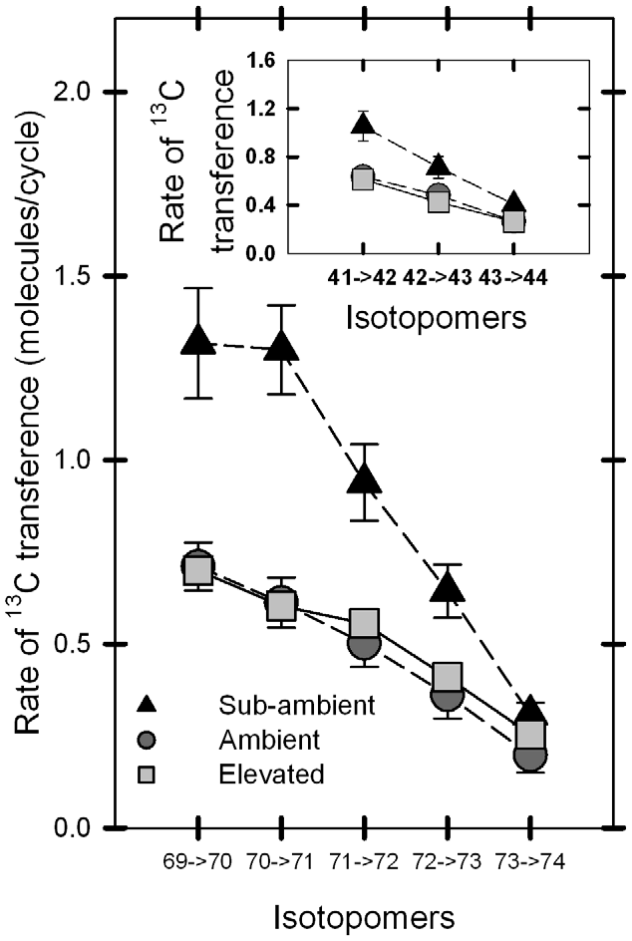
Rates of ^13^C transference between isotopomers as a function of CO_2_ concentrations. Mean rates of loss (mean ± SEM) of the labeled isotopomers in units of molecules/cycle (cycle = detection every 30 seconds with a PTR-MS dwell time of 2 seconds) for both the parent molecule M69^+^ and its fragment M41^+^ (inset graph) among individuals grown at three different CO_2_ concentrations (sub-ambient = 190 ppm (black triangles; dashed line); ambient = 400 ppm (dark gray circles; dashed line); elevated = 590 ppm (light gray squares; solid line)). In general, the photosynthetic pools of the leaves grown in sub-ambient CO_2_ were labeled faster than leaves grown at ambient or elevated CO_2_.

Because on-line PTR-MS can distinguish individually labeled isoprene species during ^13^C labeling, we measured the rates at which each mass variant appeared and reached steady state. This, in turn, allowed us to estimate the rates of ^13^C transferred from ^13^C-labeled photosynthate into isoprene as the rate of mass loss from the M69^+^, M70^+^, M71^+^, M72^+^ and M73^+^ signals. The rate of transfer of ^13^C into isoprene was ∼2 times faster for the first four masses in the leaves of poplars grown and measured under sub-ambient CO_2_ conditions, compared to those grown and measured under ambient and elevated CO_2_ conditions (Fig. 4). The rate of transfer showed the same trend in the loss of M73^+^, compared to the other masses, but the trend was not statistically significant. Similarly, the rate of mass loss for M41^+^, M42^+^ and M43^+^ was approximately twice as fast for the leaves grown under sub-ambient CO_2_, compared to the other two treatments (Fig. 4, inset). Trees grown under sub-ambient CO_2_ exhibited net CO_2_ assimilation rates ∼2 times lower than trees grown under elevated CO_2_ as shown (Fig. 1A), despite having higher stomatal conductance rates (Fig. 1C). Thus, recently assimilated ^13^CO_2_ was transferred at a greater rate into isoprene in leaves grown under sub-ambient CO_2_ compared to leaves grown under elevated or ambient CO_2_, despite having lower net CO_2_ assimilation rates.

**Figure 5:**
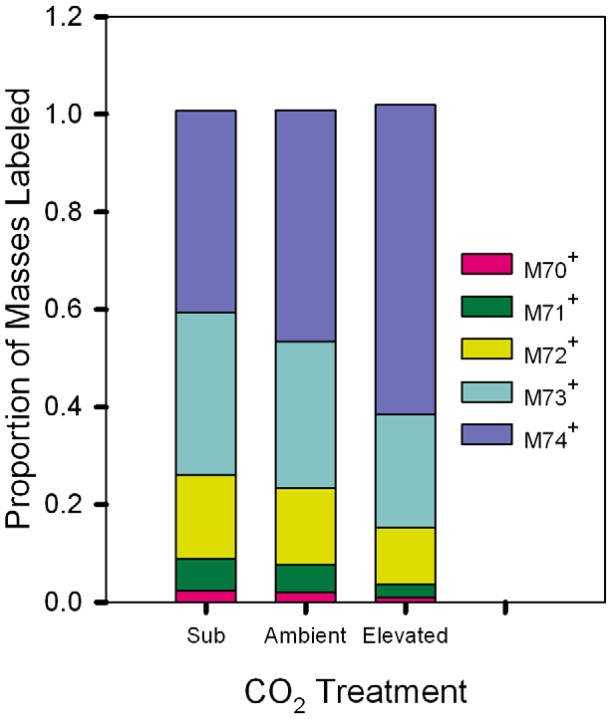
Proportion of isotopomers of the parent isoprene molecule labeled at the conclusion of the experiment. The mean proportion of the isotopomers of the parent isoprene molecule labeled at the conclusion of the experiment. Values were taken at stabilized conditions after ∼2 hr. Leaves grown in sub-ambient CO_2_ demonstrated significantly lower proportions of total ^13^C labeling (M74^+^) compared to the high proportion of total labeled isoprene molecules from leaves grown in elevated CO_2_.

The slope data illustrate how quickly pools contributing carbon to isoprene production became labeled (Fig. 4); however, it is also necessary to consider the proportion of each mass that becomes completely labeled (Fig. 5). While carbon pools that contributed to isoprene production in trees grown under sub-ambient CO_2_ exhibited the fastest initial rates of labeling and reached steady state more quickly than trees grown under higher CO_2_, sub-ambient CO_2_ trees also had the lowest proportion of total isoprene molecules completely labeled (0.413±0.026, n = 7, Fig. 5 M74^+^), a value only two-thirds that for trees grown under elevated CO_2_ (0.635±0.014, n = 7, *P*<0.0001, Fig. 5 M74^+^). Furthermore, trees grown under ambient CO_2_ had significantly less isoprene completely labeled (0.474±0.040, n = 5, *P*<0.01, Fig. 5 M74^+^) compared to elevated CO_2_ trees, but were not significantly different from trees grown under sub-ambient CO_2_ (*P* = 0.302). A very similar pattern of labeling was also demonstrated by the methyl-vinyl fragment where trees grown under sub-ambient CO_2_ had the lowest proportion of total fragment molecules completely labeled (0.587±0.022, n = 7), and this proportion was about three-fourths of that for trees grown under elevated CO_2_ (0.767±0.029, n = 7). Also similar to labeling occurring in the parent molecule, trees grown under ambient CO_2_ had significantly less isoprene completely labeled (0.634±0.031, n = 7, *P*<0.05) compared to elevated CO_2_ trees, but were not significantly different from trees grown under sub-ambient CO_2_ (*P* = 0.474).

### Total isoprene emission rates

**Figure 6:**
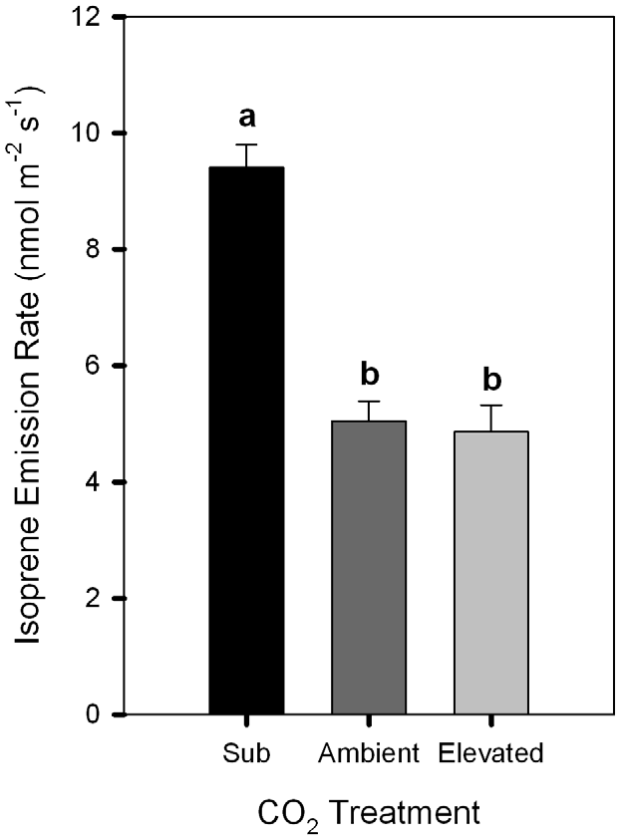
Isoprene emission rates for poplars grown under 3 different CO_2_ regimes. Mean isoprene emission rates measured as the total of M69^+^ prior to the labeling experiment for poplars grown under 3 different CO_2_ regimes (sub-ambient = 190 ppm (black bar); ambient = 400 ppm (dark gray bar); elevated = 590 ppm (light gray bar)). Error bars represent the standard errors of the mean (SEM) and means with the same letter are not significantly different (*P*≤0.05). Trees grown in sub-ambient CO_2_ demonstrated significantly higher isoprene emission rates compared to trees exposed to ambient and elevated CO_2_ concentrations.

Before the labeling treatment, we measured isoprene emissions of poplar leaves from trees grown under all three CO_2_ regimes. While the total isoprene emission rates between trees grown under ambient and elevated CO_2_ were not significantly different (n = 7, *P* = 0.946), the trees grown under sub-ambient CO_2_ had significantly higher isoprene emission rates (9.41±0.39 nmol m^−2^ s^−1^) than rates exhibited by trees grown under ambient (5.04±0.35 nmol m^−2^ s^−1^; n = 5, *P*<0.01) or elevated (4.86±0.46 nmol m^−2^ s^−1^; n = 7, *P*<0.001) CO_2_ (Fig. 6).

## Discussion

The goal of our study was to provide insight into the regulatory mechanisms controlling isoprene production, particularly the contribution of carbon from recently-assimilated CO_2_. The tracking of recently-assimilated CO_2_ into isoprene biosynthesis has been accomplished in past studies using gas chromatography-mass spectrometry (GC-MS) [32], as well as the PTR-MS approach [18], [21]. However, our study is the first to apply the PTR-MS approach to the issue of how carbon allocation to isoprene emission changes when trees are grown at different atmospheric CO_2_, including a level expected to be reached in the next few decades due to continued fossil fuel burning. Furthermore, we aimed to clarify the potential roles of pyruvate compared with G3P as substrates that control the response of isoprene biosynthesis in different growth and measurement CO_2_ concentrations. We highlight two major conclusions: (1) When the rate of photosynthetic assimilation of atmospheric CO_2_ decreases, due to limited availability of CO_2_, emitted isoprene molecules show more evidence of biosynthetic construction from stored (older) carbon sources than from recently-produced photosynthate. This is consistent with the results from Funk et al. (2004), though in that case net CO_2_ assimilation was limited by severe water and temperature stress [20]. (2) The flow of carbon from alternative, older sources most likely enters the MEP pathway through the pyruvate substrate, rather than the G3P substrate. This latter conclusion provides insight into the central role of cytosolic PEP as a control point for the channeling of carbon from different sources into isoprene biosynthesis. In the next few paragraphs, we expand on each of these two points.

Poplar leaves grown under elevated CO_2_ emitted a significantly higher fraction of isoprene molecules completely labeled with recently assimilated ^13^C than did leaves grown under ambient or sub-ambient CO_2_. This is consistent with the significantly higher internal CO_2_ concentrations and carbon assimilation rates found in poplars grown under elevated CO_2_ conditions. Furthermore, carbon pools contributing to isoprene biosynthesis were labeled ∼2 times slower in trees grown under elevated CO_2_ conditions such that these trees displayed the lowest isoprene emission rates and slowest initial labeling, but had the largest proportion of isoprene completely labeled at steady-state. We interpret these results as indicating that the rate of carbon utilization for isoprene production was relatively low in the trees grown at elevated CO_2_, allowing the availability of photosynthate produced from recently-assimilated ^13^CO_2_ to be closer to the margin required to support that low utilization rate, compared to trees grown under sub-ambient CO_2_. In trees grown under sub-ambient CO_2_ where the rate of carbon utilization for isoprene was low, the availability of recently assimilated ^13^CO_2_ was likely significantly below the margin required to support isoprene emission. This would have forced greater reliance on older, stored carbon substrate.

An alternative explanation for the decrease in ^13^C labeling of emitted isoprene from leaves grown at sub-ambient CO_2_ is that there is always some level of incomplete labeling of photosynthetic intermediates in the pentose phosphate pathway. It has been known for many years that intermediate compounds in the photosynthetic carbon reduction cycle can approach an asymptote of approximately 90% labeling when exposed to labeled atmospheric CO_2_, and this was shown most recently in the study by Hasunuma et al. (2010) using *Nicotiana tabacum* leaves [33]. Past studies using nuclear magnetic resonance, accompanied with ^13^CO_2_ labeling of soybean leaves, have shown that dilution of the ^13^C-labeled PGA pool by unlabeled glycerate from photorespiration causes a delay in full ^13^C labeling of photosynthetic metabolites [34]. However, this study also showed that the lag was brief, and that by 8 minutes after switching from ^12^CO_2_ to ^13^CO_2_, photosynthetic metabolites were 95% labeled. In our present study, only 41% of the emitted isoprene from leaves grown under sub-ambient CO_2_ was *completely* labeled after two hours of exposure to ^13^CO_2_. While it is possible that this lack of complete labeling in isoprene could be due to unlabeled G3P which originates from unlabeled intermediates of the photosynthetic carbon reduction cycle and subsequently enters the MEP pathway, this explanation would not account for the fact that the greatest fraction of incomplete labeling in the isoprene molecule occurs in the fragment derived from pyruvate. Logic would lead us to expect that a carry-over of incomplete labeling from the photosynthetic carbon reduction cycle should show up in the G3P derived fragment of isoprene first. This is not the case. The simplest explanation is that there exists a constant channeling of unlabeled, older stored carbon into isoprene biosynthesis, through the pyruvate substrate, which dilutes the ^13^C-labeling of isoprene to a steady-state value that varies as a function of CO_2_ availability. The source of this stored carbon remains to be identified.

Our results suggest that the C-1 carbon of G3P, which would be the first to be labeled after assimilation of ^13^CO_2_, is transferred quickly to isoprene and thus appears quickly as C-1 of the methyl-vinyl fragment. This carbon presumably enters isoprene from chloroplastic G3P that moves directly into the MEP pathway. Following the entry of ^13^C through the C-1 of G3P, the label appears to enter isoprene through the C-2 and C-3 of G3P, as evidenced by the progressive divergence of the M41^+^ line from the M69^+^ line. We interpret the slow and incomplete labeling of the methyl-vinyl fragment to indicate the carbon derived from pyruvate carry the label into isoprene even more slowly than the carbons of G3P, and that some fraction of these carbons are perpetually derived from older, unlabeled stored carbon sources. Our observation of an inverse relationship between atmospheric CO_2_ growth and measurement conditions and isoprene synthesis in poplar leaves is in agreement with a number of other studies, particularly for trees grown at elevated and sub-ambient CO_2_ concentrations [10], [23], [24], [27]. Our results showed that isoprene emission rates observed from poplars grown under ambient and elevated CO_2_ were statistically equivalent. It is likely that our experiment suffered from an inadequate sample size required to resolve differences beyond sample-to-sample variance, though the trends are consistent with what we might expect. The cause of this anti-correlation has been suggested to be the down-regulation of isoprene synthase activity when trees are grown in the presence of elevated CO_2_ [35] and/or the up-regulation of cytosolic PEP carboxylase in the case of growth at elevated CO_2_ or increase in activity of PEP carboxylase in the case of short-term measurements at elevated CO_2_ [24], [27].

**Figure 7:**
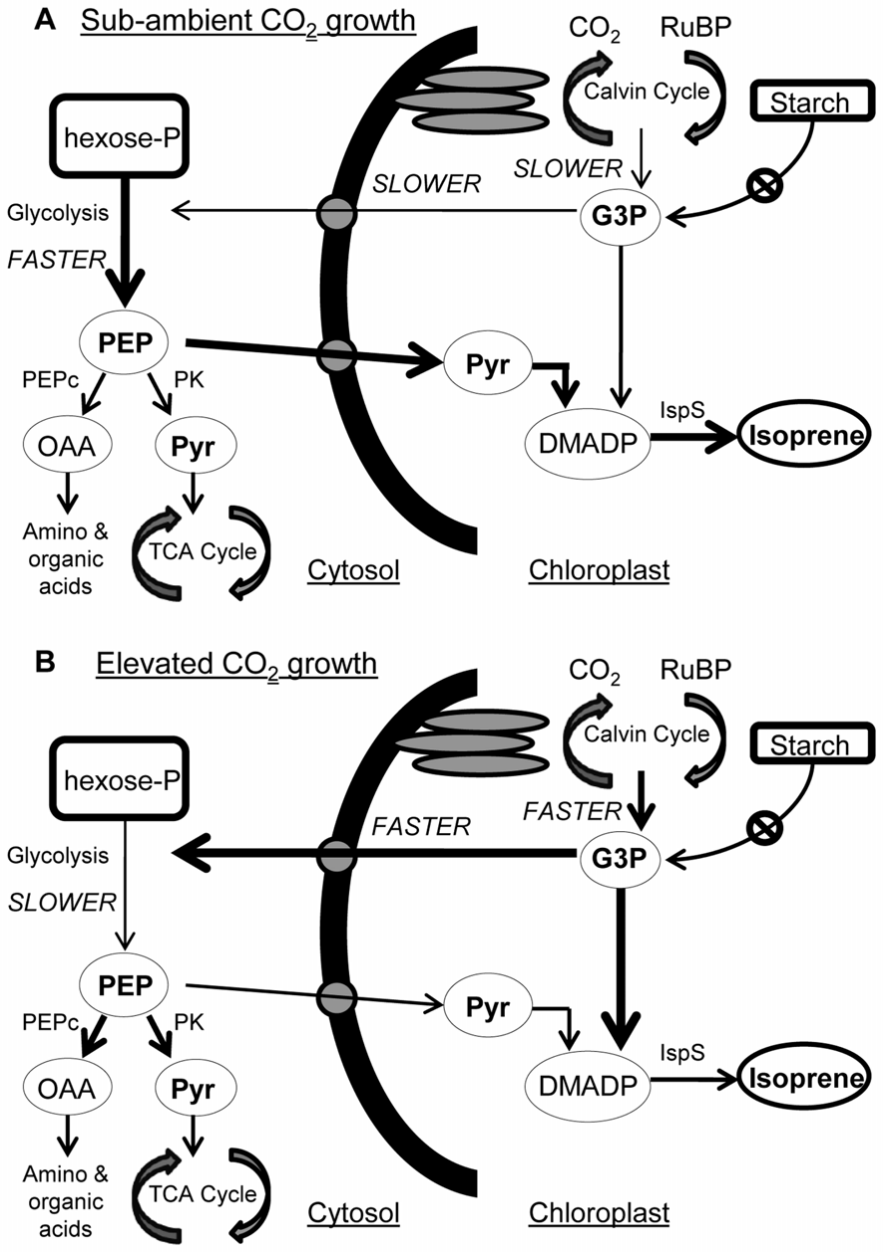
Conceptual model illustrating the flow of carbon contributing to isoprene synthesis. Conceptual model illustrating the flow of carbon contributing to isoprene synthesis from both recently assimilated carbon in the form of glyceraldehyde-3-phosphate (G3P), chloroplastic carbon sources (starch), and extra-chloroplastic carbon (hexose phosphate) via glycolysis and the production of phospho*enol*pyruvate (PEP) under sub-ambient (A) and elevated (B) CO_2_ growth and measurement conditions. Arrow thickness designates rates of production or transport. Low CO_2_ availability (panel A), compared to higher CO_2_ availability (panel B) result in substrate limitations to isoprene synthesis because of slower rates of G3P production and reduced/negligible availability of stored starch. Furthermore, no starch is thought to be contributing to isoprene synthesis under elevated CO_2_ conditions as simultaneous breakdown and synthesis is yet to be shown in poplar and the stored carbon utilized for isoprene synthesis is thought to come from extra-chloroplastic sources. RuBP = ribulose 1,5-bisphosphate; OAA = oxaloacetate; Pyr = pyruvate; TCA = tricarboxylic acid; DMADP = dimethylallyl pyrophosphate; PEPc = phospho*enol*pyruvate carboxylase; PK = pyruvate kinase; IspS = isoprene synthase.

Given our conclusion that the isoprene carbon derived from older, stored reserves is channeled through the Pyr substrate, we can begin to piece together a conceptual model based on our hypothesis for how the differential control of isoprene emission by CO_2_ availability might occur. In our model, low CO_2_ availability compared to higher CO_2_ availability would generally result in substrate limitations to isoprene biosynthesis, particularly DMADP, because of slower rates of G3P production and reduced/negligible availability of stored starch (Fig. 7A and 7B for low- and high-CO_2_ availability, respectively). Furthermore, no starch is thought to be contributing to isoprene synthesis under elevated CO_2_ conditions as the simultaneous breakdown and synthesis of these storage carbohydrate structures remains undocumented in poplar [19]. Assuming that the demand for chloroplastic pyruvate remains relatively high in the face of these substrate limitations, and that the flux of G3P through glycolysis is regulated to be nearly constant [36], then the gap between the availability of recent photosynthate/starch and substrate demands of the MEP pathway may be closed by the mobilization of extra-chloroplastic carbohydrate reserves.

Homeostatic maintenance of the glycolytic flux in the face of reduced sugar availability has been demonstrated in tomato cell cultures [36], and in the case of our model would be required to maintain the production of pyruvate substrate from mobilized, extra-chloroplastic carbon sources. This type of regulation on the supply side of PEP production may be augmented by changes in the demand for pyruvate in the chloroplast due to up-regulation of MEP pathway or isoprene synthase gene expression when plants are grown under sub-ambient CO_2_. Up-regulation may occur if end products of the MEP pathway (e.g., carotenoids, abscisic acid (ABA), or isoprene itself) are needed to enhance tolerance of the stresses imposed by a constrained net CO_2_ assimilation rate. In that case, the mobilization of alternative carbon sources may be triggered by increased demand for pyruvate substrate to drive amplified MEP pathway activity, and that increased demand could be met with extra-chloroplastic or chloroplastic sources of stored carbohydrate. The gap between demand for Pyr substrate to synthesize isoprene, and what can be provided through cytosolic processing of recently assimilated photosynthate, was clearly observed in our experiments in the comparison between the leaves measured at sub-ambient CO_2_ and those measured at elevated CO_2_ (i.e., the extremes of the treatments). In that case, leaves from the sub-ambient treatment exhibited both lower fractions of total isoprene and three-carbon fragment that were labeled with ^13^C, and higher isoprene emission rates, compared to leaves measured at elevated CO_2_. The leaves measured at ambient CO_2_ were not clearly distinguishable from leaves measured in the sub-ambient and elevated CO_2_ treatments.

Notably, Rasulov and co-workers have explained the CO_2_ response of isoprene emission in terms of limitations of chloroplastic ATP, rather than the import of cytosolic PEP [37], [38]. If the limitation to isoprene biosynthesis rate were solely due to chloroplastic ATP at elevated CO_2_, rather than availability of Pyr substrate, the differences in the labeling kinetics we observed between treatments simply cannot be explained. Limited ATP availability at elevated CO_2_ imposed by reduced inorganic phosphate (Pi) could indeed explain reduced isoprene emission rates. However, if ATP availability was the ultimate control over the CO_2_-sensitivity of isoprene emission, then the proportion of ^13^C label in the isoprene emitted from leaves measured at sub-ambient CO_2_ would be similar to the isoprene emitted from leaves at elevated CO_2_, which is not what we observed.

### Conclusions

Poplar trees grown under sub-ambient CO_2_ exhibited higher isoprene emission rates with a higher proportion of incompletely-labeled isoprene. Across all CO_2_ treatments, the first carbon that contributed to isoprene synthesis appears to be derived from a rapidly labeled G3P pool, while the last two carbons come from a more slowly labeled pyruvate source. The fact that all treatments showed some level of incomplete labeling suggests that the carbon that goes into making pyruvate comes at least partly from older carbon sources within the plant. Overall, we conclude: 1) that trees experiencing low photosynthetic rates due to reduced atmospheric CO_2_ availability have a higher percentage of carbon from stored/older carbon sources for isoprene biosynthesis, 2) that carbon most likely enters the MEP pathway through the pyruvate substrate, and 3) that extra-chloroplastic rather than chloroplastic carbon sources are most likely mobilized to increase the availability of pyruvate required to support an up-regulation of the MEP pathway.

Our study shows that trees grown under conditions that limit CO_2_ assimilation rely more heavily on extra-chloroplastic carbon sources, most likely via the pyruvate substrate, for isoprene biosynthesis. However, the identities of these alternative carbon sources, their relative importance under long-term exposure to altered atmospheric CO_2_ concentrations, and the specific role of pyruvate, remain unknown. Starch is often suggested as a possible carbon source for isoprene synthesis, particularly under elevated CO_2_ conditions. To discriminate between starch degradation and alternative extra-chloroplastic sources in providing carbon for isoprene production, long-term CO_2_ studies using isotopic labels coupled with PTR-MS methodologies are needed. Starch accumulation could be quantified over time in trees grown under various CO_2_ regimes and the relative contribution of those labeled carbons toward isoprene synthesis would be assessed using methods similar to those described above. While starch does not appear to play a role in short-term carbon contribution towards isoprene synthesis—particularly in our study—this relationship may change under long-term exposure to altered atmospheric CO_2_ with subsequent changes in carbon allocation dynamics as the plants acclimate. In addition to long-term studies, future work should also consider following isotopes through other potential carbon contributors toward isoprene synthesis to identify these elusive carbon sources, although this may prove difficult considering the exchange rate of chloroplastic and extra-chloroplastic carbon compounds. Large sample sizes and considerable investment are likely required for a more comprehensive analysis of carbon allocation and isoprene biosynthesis.
